# “My life became more meaningful”: confronting one’s own end of life and its effects on well-being—a qualitative study

**DOI:** 10.1186/s12904-022-00950-3

**Published:** 2022-04-29

**Authors:** Helena Kukla, Angélique Herrler, Julia Strupp, Raymond Voltz

**Affiliations:** 1 Faculty of Human Sciences and Faculty of Medicine, Graduate School GROW – Gerontological Research on Well-being, University of Cologne, Albertus-Magnus-Platz, 50931 Cologne, Germany; 2 Faculty of Medicine and University Hospital, Department of Palliative Medicine, University of Cologne, Cologne, Germany; 3 Faculty of Medicine and University Hospital, Center for Integrated Oncology Aachen Bonn Cologne Duesseldorf (CIO ABCD), University of Cologne, Cologne, Germany; 4 Faculty of Medicine and University Hospital, Clinical Trials Center Cologne (ZKS), University of Cologne, Cologne, Germany; 5 Faculty of Medicine and University Hospital, Center for Health Services Research (ZVFK), University of Cologne, Cologne, Germany

**Keywords:** End of life, Well-being, Quality of life, Palliative care, Aged, 80 and over, Coping, Attitude to death, Spiritual care, Qualitative research, Terminally ill, Finitude, Psychosocial support system

## Abstract

**Background:**

The perception of being closer to death can be experienced due to old age or life-limiting diseases, and can pose profound existential challenges. Actively confronting death-related issues and existential questions may increase psychosocial comfort and stimulate personal growth, whereas dysfunctional coping may lead to existential distress.

To date, research on individual and (semi-)professional approaches to confronting the own end of life and the effects on one’s well-being remain scarce. Therefore, the aim of this study was to explore individual strategies and wishes in order to derive ideas for appropriate support concepts.

**Methods:**

Twenty semi-structured interviews were conducted with people over the age of 80 (*n* = 11) and with a life-limiting disease (*n* = 10). The interviews were transcribed verbatim and independently coded by two researchers according to Braun and Clarke's thematic analysis approach.

**Results:**

While the use of (semi-)professional approaches (e.g., therapeutic support) to confronting existential questions in the shape of one’s impending death was rare, individual coping strategies did have a positive impact on psychosocial comfort. There were hardly any significant differences between the participants aged 80 and over and those with a life-limiting disease in terms of individual coping strategies or how they approached the ends of their lives. Both groups reported that theoretical education, preparing for the ends of their lives (e.g., funerals), talking about death-related topics, reflecting on death-related topics, and contemplating death in a spiritual sense had positive effects on their assurance, self-determination and relief. The necessity of confrontation and a desire for low-threshold, accessible and flexible services to meet their existential and spiritual needs were highlighted.

**Conclusions:**

There is both a desire and a need for the addressing of existential questions. Outside of private contexts, however, the participants possessed little awareness of support services that focused on confronting end-of-life issues, and rarely used such services. Efforts to raise awareness for psychosocial and spiritual needs should be implemented within the care system, together with low-threshold support concepts, in order to increase psychosocial well-being. More research evaluating individual approaches to confronting the own end of life are needed to better understand this determinant of well-being and its mechanisms of action.

**Trial registration:**

www.germanctr.de, DRKS*-ID*: DRKS00020577.

**Supplementary Information:**

The online version contains supplementary material available at 10.1186/s12904-022-00950-3.

## Background

Natural aging processes and life-limiting diseases can be associated with declines in physical and mental competence, and result in an increasing awareness of the end of one’s life [1–5]. Age and disease-related changes trigger the subjective perception of being close to death, and are therefore associated with mortality salience [6, 7]. The prominent Terror Management Theory addresses the pervasive role of the awareness of one’s own end of life, which influences the psychological well-being. This can lead to high levels of discomfort and anxiety, rooted in the existential dilemma that death will ultimately occur [7]. Physical and mental losses and the related awareness of the impending death usually trigger existential questions that focus on the following themes described by Frankl and Yalom amongst others [8, 9]: finding meaning and meaning stagnation including issues of life and death and questions of the value of life connectedness, hope and anxiety [8–12]. These themes can be allocated to the overarching category “struggle to maintain self-identity” [12]. This challenging phase of life may cause existential suffering. According to the popular model of total pain concept of the 1960s, Cicely Saunders stresses the multidimensionality of pain and considers existential suffering as a result of meaninglessness [13]. The intactness of a person is threatened [14]. Meanwhile, the former connection between physical pain and existential suffering is debated intensively, but the existence of existential suffering is uncontroversial and perceived as relevant in health care settings [15]. However, research how to understand, manage and treat existential issues is needed [10, 16].

Consciousness of the impending end of one’s life and therefore a lack of coherence and orientation requires a process of psychological adjustment of the self [10, 17, 18]. A resistance to change and the avoidance of one’s finitude can result in dysfunctional coping strategies and increase distress [5, 7, 19], whereas an accepting attitude can decrease anxieties and help to ensure a good death [20, 21]. Current research shows that confronting death-related topics can also stave off distress and even provide capacity for personal growth [22, 23]. The process of adaptation enables individuals to progress toward an accepting attitude to death [6, 24].

The connection to spirituality, which is defined as the act of expressing and seeking meaning, purpose, transcendence and connections to others, oneself and a higher power, can be a key aid in adjusting to the own end of life. Psychosocial and spiritual needs come to the fore alongside physical needs [10, 25–28] and are often interconnected with existential needs [29] and may even play the most important role within the field of healthcare [30]. The individual needs may neither be clearly assigned to spiritual nor existential dimensions, but are often intertwined [31]. Hvidt et al. [32] consider that “the existential” could represent an overarching concept including spiritual meaning orientations, but focusing less on transcendent aspects.

The need for psychosocial and spiritual care, that addresses the needs of people in their search for meaning in life, often remains unmet [30, 33–35]. The assessment and treatment of spiritual problems is established within the field of palliative care, but is given too little consideration within healthcare in old age and research [36]. In order to address individuals’ uncertainties and their feelings towards death, dying and existential questions, person-centered holistic care must be provided [28, 37]. In other European countries, such as the Netherlands and the United Kingdom, spiritual care plays an essential role, and is therefore more easily accessible [37–39]. In Germany, however, spiritual and psychosocial support needs to be anchored more firmly in healthcare setting, and consequently in education for healthcare practitioners [40, 41]. Hence, there is a need to promote research, that includes spiritual needs, assessment and treatment of spiritual distress.

Although existential symptoms and spiritual distress are among some of the most debilitating issues, approaches to confronting these difficult demands and the underlying mechanisms of action have received little attention in research [10, 42, 43]. In particular, research based on individuals who are concerned with their impending death from a first-order perspective is generally under-represented [1, 2].

Previous research is based mainly on professional psychosocial approaches to confronting finitude, and contemplates the aforementioned issues to some extent, stating predominantly positive effects on psychosocial well-being that include relief, self-determination and assurance [44, 45]. Focusing on spiritual care, i.e., addressing uncertainties and feelings toward death and dying, as well as existential questions [28], can improve an individual’s psycho-emotional stabilization, strengthen their self-identity and sense of purpose, and promote adjustment to death [17, 30, 43, 46]. Consequently, strategies to respond to these healthcare challenges unique to the end of life are needed in order to preserve quality of life for those nearing death.

The individual coping strategies and little-used (semi-)professional support concepts, which are understood as short-term interventions provided by trained personnel in some healthcare fields (e.g., nurses, psychologists), chaplains or skilled, trained volunteers, require further investigation and evaluation. As such, the aim of this qualitative study is to explore how people aged 80 and over or who have a life-limiting disease confront the end of their own lives, and how they experienced the effects of doing so. Wishes regarding the confrontation of one’s finitude and potentially appropriate forms of intervention will be derived based on the results.

## Methods

This qualitative study was conducted within the Graduate School GROW – Gerontological Research of Well-Being at the University of Cologne. The reporting is based on the Consolidated Criteria for Reporting Qualitative Research (COREQ) checklist. Ethical approval was obtained from the Ethics Committee of the University of Cologne (approval no. 19–1617).

### Participants

The study focused on the section of the population with increased proximity to death due to old age or the diagnosis of a life-limiting disease. As a result, people aged 80 and over and people with a clinician-diagnosed life-limiting disease (no reasonable hope of cure and likely to cause premature death, e.g., cancer, heart diseases, neurodegenerative diseases) and able to conduct the interview were included in the study. People with cognitive impairments or limited ability to communicate were excluded.

Participants were selected purposely. Potential participants were approached with the help of healthcare stakeholders involved in the general care for the selected participants. These acted as entry points by recommending other stakeholders, and provided important both verbal and written information on the aim of the study, structural contexts and informed consent. In addition to this, participants were recruited via online channels, such as forums, email lists and newsletters. Participants were recruited until meaning saturation was reached and no new ideas or issues arose [47], i.e., the point where all the conceptual issues had been captured and the interviewees’ meaning and insights were fully understood [48, 49]. We monitored the depth of data by documenting any new dimensions or insights of issues. After eight interviews and subsequent discussion, the conceptual scope of themes and codes was saturated, indicating that changes in the content of the codebook were completed. In order to establish sufficiency of conceptual depth and richness and diversity of data, we conducted and analyzed further interviews [50, 51].

### Study procedure and data collection

We developed an interview guide based on existing literature and a previous literature review [44]. The interview guide was discussed within the research team and pilot tested with one participant to ensure clarity and wording of questions.

The interview guide (see Table 1) was used to encourage participants to tell their personal stories and death-related experiences, as well as individual confrontation with their own finitude and wishes for opportunities to confront existential questions. The semi-structured design allowed us to be responsive to in-depth personal accounts using sub-questions intending to elaborate beyond the participant’s initial response and rephrasing questions to elicit the pertinent information [52]. In order to convey meaning the interviewer diverged slightly from the script and motivated the participants to provide detail and clarifications. We adapted the interview guide in light of the current Sars-CoV-2-situation, and added two questions relating to changes of attitudes due to the pandemic.

**Table 1:** Interview guide

Block I: Experiences
We’re going to be talking today about the topic of death and dying. What experiences have you already had with death and dying?
What thoughts do you have when you think about the end of your own life?
How have you dealt with the finitude of your own life?
Having told us how you have dealt with this topic, what effect did this process have on you?
Recently, our lives have been marked by the Sars-CoV-2 pandemic, also known as Covid-19. I would like to talk to you about this. To what extent have you found yourself dealing with the topics of death and dying, especially during the Sars-CoV-2 pandemic?
To what extent have your attitudes towards dying and death changed as a result of the Sars-CoV-2 pandemic?
Block II: Support for dealing with the issue
What specific support or services would you like to receive in order to confront the topics of dying and death?
Block III: Conclusion
Is there anything else you would like to discuss or say on this topic?

In an initial conversation via telephone, the locations and scheduling for the face-to-face semi-structured interviews were chosen by the participants. The first author (HK), experienced in the field of end-of-life conversations particularly in hospice settings, provided information on the study, the interviewers background and pseudonymized data processing prior to the interviews. The participants were reassured that the interviews would be confidential and voluntary. Written, informed consent was obtained. Field notes were taken during the interview.

The audio of the interviews was recorded and transcribed verbatim using an external transcription service. The transcripts were checked by the first author and pseudonymized prior to analysis, and stored on a secure server. We provided the transcripts and results of the study to the participants on request.

### Data analysis

Two experienced researchers (HK, speech therapist, and AH, health scientist), both of whom are experienced in qualitative data analysis, analyzed a core set of interviews using thematic analysis by Braun and Clarke [53]. This approach is an inductive method that allows emerging codes (single ideas associated with a segment of data) and themes (patterns of shared meaning) to be grounded in the original data. Both researchers were familiar with the setting and AH was given information about the context of the interview including surrounding, initial conversations and perceived mood of the participants in a written form. Using a six-phase model (see Table 2), the transcripts were analyzed independently: [1] After familiarizing themselves with the data and forming initial analytical observations, HK and AH [2] independently coded a set of four interviews line-by-line. [3] Similarities and differences in codings were discussed and sorted into themes independently until a consensual codebook was developed. [4] Four further transcripts were then analyzed independently on the basis of this codebook in order to review and modify the themes and related codes. [5] After the differences between the two authors had been merged, a final version of the codebook was created as a result. This point was reached [6] Before the themes were interconnected and contextualized with the current state of research, the codebook underwent a final validation within the research team. After analyzing 50% of the transcripts independently and reaching data saturation the revised codebook was completed. Subsequently, all interviews were fully coded by HK based on the codebook. The coding of ambiguous parts in the interviews was double-checked independently with the second author (AH) and discussed until a consensus was reached [53].

**Table 2:** Analysis procedure according to Braun and Clarke [53]

Steps	Procedure	Example
1. Familiarization	HK & AH independently: Reading and rereading the transcripts and noting down initial codes	Citation: *I think I have to act more responsible and forward-looking concerning the end of life. What keeps me busy is the elimination of things that I collected. That is my priority at the moment* (Interview 4: A, pos. 42) Initial codes: priorities before dying (HK), focus on essentials in life (AH)
2. Generating initial codes	HK & AH independently: Inductive coding line-by-line of four interviews	Independent coding: get things done (HK), before dying (AH)
3. Searching for themes	HK & AH independently: Checking and discussing codes for redundancies and sorting into themes	Related themes: feelings and attitudes to death (HK), the individual way until death (AH)
4. Reviewing themes	HK & AH independently: Modifying themes and generating a thematic map on the basis of four further interviews	Merged and modified theme: Before dying and agreed final code name: get things done
5. Defining and naming themes	HK & AH: Merging differences a final codebook was created and reviewed by AH	Discussing the revision of the codebook with AH and checking the related codings
6. Coding and reporting	Research team: Validating the final version of the codebook and independent coding of ambiguous parts of interviews by AH and HK	Proof or refinements of the whole research team

## Results

21 Interviews (*n* = 11 people aged 80 or older and *n* = 10 people with a life-limiting disease) were conducted between August 2020 and June 2021, lasting between 25 and 98 min each (see Table 3 for information on participants). The interviews were conducted in private without distractions, and took place in each participant’s location of choice: at the participant’s home (*n* = 16), in a hospice (*n* = 3), in a care home (*n* = 2) or on the premises of the University (*n* = 1). In one case, the son of the interviewee was present. 11 main themes and 66 subthemes were identified (see Additional file 1).

**Table 3:** Information on participants

Number	Age	Gender	Participants aged 80 and over (A) and people with a life-limiting disease (D)	Setting
1	90	Female	D (cancer)	Home
2	89	Female	A	Senior care home
3	81	Female	A	Home
4	91	Male	A	Home
5	87	Female	D (cancer)	Hospice
6	88	Female	A	Home
7	80	Female	D (cancer)	Home
8	84	Female	A	Home
9	81	Male	A	Home
10	78	Female	D (cancer)	Home
11	83	Male	A	Home
12	97	Male	A	Home
13	87	Female	A	Senior care home
14	91	Male	A	Home
15	81	Female	A	Home
16	58	Male	D (cancer)	University
17	80	Female	D (cancer)	Hospice
18	70	Male	D (cancer)	Home
19	66	Female	D (cancer)	Hospice
20	78	Female	D (cancer)	Home
21	68	Male	D (cancer)	Home

### Differences between people aged 80 and over and people with a life-limiting disease

No significant differences were identified between the two participant groups in terms of their approaches to confronting the end of their lives, their initiative and motivation to engage in confrontation, or their individual wishes for support in this area. In both groups, the participants who displayed more of a dismissive attitude did not necessarily avoid death-related topics; indeed, some of them explicitly mentioned the necessity of discussing these issues. For example, one participant avoided any contemplation of his life-limiting diagnosis: “*So if someone says, yes, you are running away from it, then I think, yes, you could be right, you can see it that way*.” *(Interview 16: D, pos. 66)*. However, he expressed an ambivalence toward his own perspectives *“But that doesn’t help, you have to talk about it. Otherwise, I don’t know, then it will somehow get lost, because the process is not under control.” (Interview 16: D, pos. 128)*. Within this interview setting, the subjective perception of being close to death seemed to be comparable between the two groups. According to the participants, their attitudes toward death have not changed as a result of the pandemic, and most of them were cautious to avoid infection and the risk of dying from Sars-CoV-2.

### Approaches to confronting the own end of one’s life and their effects

In their narrations, the participants described a variety of approaches to confronting the own end of life: theoretical education, preparing for one’s own death, talking about death-related topics, reflecting and thinking about death-related topics, and faith or and spirituality-based approaches.

#### Theoretical education

In both groups, theoretical knowledge regarding death and dying was often acquired through readings or literature. In some cases, the participants had also undergone further education on the topic on their own initiative. Theoretical education included films, Internet research with a potential focus on recent research results, and information sources such as physicians and specialized outpatient palliative care. This form of confrontation was usually chosen by the participants themselves, and often led to assurance. This theoretical knowledge growth resulted in new coping strategies and the development of additional points of reference for the final phase of the patient’s life.

*To find out what intellectual people have thought and written about it can lead to personal growth. (Interview 6: A, pos. 133)*

*So I found that very important, to put myself into context with other individuals. And not to think that things happen to me alone, they (…) are to do with development or with stages of life. (Interview 6: A, pos. 133)*

The described coping strategy in this difficult phase of life indicated that scientific knowledge has the power to provide certainty. The overall positive effects also included the relieving realization that others were in a similar situation, as one participant concluded.

*Ageing research (…) I find that relieving and reassuring, to see that it is part of it and that’s how it is, and for you too. (Interview 6: A, pos. 137)*

#### Preparing for death

Preparing for the end of one’s life was inevitably a confrontation with individual ideas on how to actively take control over the final phase of one’s life, and also an act of dealing with wishes for one’s estate and burial. Participants with a life-limiting disease did not take more measures to prepare for the end of life than people aged 80 and over, though in some cases they did have more concrete ideas regarding their funeral process, e.g., clothing. In the context of advance care planning, most people in both groups had drawn up a living will and described a feeling of self-determination in that they could still make their own decisions up until their deaths, and that this made them feel more secure. A sense of relief and calming effect were also achieved following the completion of advance care documents. The feeling of knowing what end-of-life processes might look like helped the participants feel less burdened. Being proactive in terms of preparing for the end of one’s life also played a role, in that many participants aimed to avoid being a social and emotional burden to others to stay as independent as possible.

*I don´t want life-prolonging measures. (…) I’ve sorted things out so far, now we just have to let things come to us. (Interview 11: A, pos. 26)*

The planning of one’s own funeral, the financing for which had also already been independently arranged in some cases, was occasionally perceived as threatening and strange, but the assurance achieved through detailed planning, up to the clothing to be worn by the deceased, also had a positive effect in two ways: On the one hand, there was a feeling that *“it’s done”* (Interview 3: A, pos. 154) and on the other hand, participants reported a sense of security that their funerals would meet their own expectations in writing.

*It was important for me to know that everything would be clean and calm and good. That those I write down are notified. (Interview 15: A, pos. 78)*

For other participants, planning was simply part of the process, and did not trigger any particular feelings.

*That’s the way it is now. There is nothing more to say. I don’t think about it all the time. That’s how it is. (Interview 6: A, pos. 37)*

#### Talking about death-related topics

The act of talking about death-related topics was mentioned frequently as a way of confronting the end of one’s life. In most cases, the participants talked to loved ones about their current situation, the feeling of being close to death due to their age or health condition, and also about their estate and funeral. Death-related topics were also discussed with friends and healthcare professionals in order to help the participants orientate themselves within the final phase of their lives, to avoid illusions, and to share their own thinking, which help them feel more light-hearted. The effects of these actions were almost exclusively described as positive; participants said the discussion was helpful and important in developing their own coping strategies and alleviating their fears.

*Yes, you then feel… well, I did, at least… you then feel a bit lighter, because he had the same thoughts as I did. And so, he says, yes, we can’t do anything about it anyway. It’s our turn. (Interview 13: A, pos. 85)*

For some participants contemplating different scenarios with others could help to come to a neutral acceptance of the own end of life. Others described discussions of death-related topics more neutral in terms of their impact, as they possessed the conviction that death was a natural process and part of life. These participants also considered a discussion of their present circumstances and emotions unexciting but unavoidable.

The participants indicated that taking part in the interviews had enabled them to gain new insights, brought forward new concepts relating to attitude, and even resulted in improved satisfaction for them.

*I’m really grateful to you for this interview, because I wasn’t aware of it at all. But it really comes more from the fact that I have integrated myself like… (longer pause), yes, into the cycles on the one hand, into the natural cycles. (Interview 19: D, pos. 60)*

Conversations could also provide clarification and relief through the building of trust between conversation partners the compassion of the other person becoming visible to the participant. This acted as a form of reassurance that the participants were "*not alone in this process of coping*." *(Interview 3: A, pos. 39)* In addition to serious conversations, humor was also perceived as a source of relief in one case.

*There is a third person living there. That’s the cancer. We named him Donald, after Trump, the ass. And if something went wrong or something, we said: I’m sorry, that was Donald. He is interfering again, the jerk. And that was helpful for me, to see it that way. (Interview 21: D, pos. 161)*

A few people said that they avoided conversations with the people closest to them, or only talked about such existential topics when the occasion arose, so as not to trigger burdensome situations themselves.

*My daughter never sees me like this. (cries) (Interview 5: A, pos. 102).*

This participant hid deep feelings and anxieties to protect her daughter from feeling responsible to handle such emotional situations but decided to carry the burden herself.

#### Personal reflections and thoughts

Part of the confrontation the participants undertook with their own was largely invisible from the outside, as it took place within the framework of the participants’ personal reflections and their own thoughts on topics such as fear of death, uncertainty regarding the future, and wishes for a dignified end of life. The participants often described feeling a sense of relief once they had established more clarity within their own thought processes and conclusions.

*I’m still too attached to life for that. But I am already thinking about how to defuse it. (…) To see it more calmly. The future of death, or what’s in store for me. I have to accept that for myself. (Interview 9: A, pos. 181–183)*

It gave the participants confidence to believe in something and to develop their owns path and attitudes. This had the potential to determine acceptance of phases of life. Participants rated personal reflections as a potential way of making life more meaningful. One unsettling effect mentioned in very rare cases was thoughts of dying during sleep.

*(…) because I think about it and don’t know what it’s like, how it happens. That scares me. (Interview 2: D, pos. 37)*

Some people described having had little exposure to the existential issues of death and dying as they had not been present in their lives, the focus instead being on life-related matters. The effects of these initial thoughts were generally perceived as neutral and served as a source of reassurance, it was commonly assumed that confronting this topic was a necessity.

#### Spirituality and faith

The participants reported turning to spirituality and faith to guide their approach to dealing with their own finitude. This approach was able to play a significant role in determining the individual’s attitude toward death and dying. Several of the participants stated that believing in God and going to church helped to reduce their fear of death and dying and increase their well-being.

*I pray to Jesus often, and the Mother of God, and I say, “You know exactly what is good for me and I put everything into your hands,” and then I have already really chosen a good number. Then I don’t need to burden anyone else. (Interview 15: A, pos. 14)*

Mechanisms of actions seemed to be based on the conviction each participant had in their own beliefs and on external guidance from a higher power; this had a facilitating effect, as it led to the belief that one can only influence the course of one’s life to a limited extent. Some people had turned away from the church without losing their faith. Spirituality was said to lead to an increase of assurance in terms of resonating with oneself and one’s beliefs. People reported a greater sense of well-being and self-determination even diving deeper into spiritual topics, and said that this enabled them to integrate death into their lives.

*Then I go into the root system of the yew tree and that feels really nice. Yes, everything seems well-rounded and that’s why I feel quiet… so especially with these ritual connections, I think that’s what really helps me to feel good. (Interview 19: D, pos. 60)*

### Need for action in confronting finitude and wishes

Some people felt that settings in which there was an opportunity to review life and to discuss death-related topics strengthened a sense of meaning. Outside of private contexts, there were not many services that provided low-threshold opportunities with a focus on confronting end-of-life issues. (Semi-)professional approaches to confrontation were difficult to access because they were not visible enough.

*It is important that this is made a bit more public – where people can find out about this, that and so on. (Interview 17: D, pos. 148)*

However, it was considered very important to remember that people are also entitled to avoid the topic, and for carers and people around those approaching the end of their lives to accept this opinion.

Therapeutic settings, including group therapy, were desired not only by people with life-limiting diseases, but also by people over 80 who wanted an honest discussion with others in their own age group. Psychological support services were requested, especially after a serious diagnosis, but there was also a desire for other forms of therapy, such as art therapy.

*Every tumor patient must be accompanied. That needs to be offered as a matter of course. (Interview 7: D, pos. 120)*

Being able to talk to one’s next of kin, people in similar situations, and psychologically trained staff or pastoral caregivers was frequently mentioned as a need. Discussion groups were rated as desirable, but here too, a lack of accessibility was expressed.

Some people showed a great deal of uncertainty regarding what services currently existed, and for many people it was thus unimaginable to take advantage of services that focus on confronting finitude.

*I don’t know. What kind of services are there? Yes, how, what can I wish for? (Interview 2: D, pos. 137–139)*

*But I don’t know where else to look. (Interview 7: D, pos. 120)*

Some did not know whether they would make use of services, others categorically refused or expressed a diffuse range of ideas and wishes regarding the implementation of such services, since the idea of what contexts in which existential questions can be addressed might look like does not allow for inferable practical implications.

## Discussion

The aim of this study was to explore perspectives on confronting death and dying among people aged 80 and over and people with a life-limiting disease. No differences were found between the two groups of participants, so it can be assumed that the subjective perception of being close to death – and thus the experiences, attitudes, issues, and the need to confront death-related topics – are comparable between both groups.

The confrontation with death and dying took place almost exclusively in private contexts rather than (semi-)professional settings, and included contemplating and talking about death-related topics, preparing for the end of one’s own life, theoretical education, and faith-based approaches. Overall, the self-reported predominantly positive effects led to an improved sense of well-being and quality of life. This validates earlier findings. Spiritual needs for control, relationships, autonomy and a sense of purpose [54] can be fulfilled. Desires among these groups for further services remained relatively unspecific, as they had no clear idea of what contexts that focus on existential questions would look like. Options for discussion in both private and professional contexts were desired.

Emotional distress, be it spiritual or psychosocial in origin, should be treated with the same importance as physical distress [17], and there was a clear need for contemplating death and addressing uncertainties and feelings surrounding this topic [28]. Addressing death-related issues can open up dialogue with people who are more reluctant to talk about these issues [55]. The present study confirms this to some extent, as some of the participants wanted to talk about topics such as preparing to die despite describing themselves as avoiders, and even felt comfortable outside of their expected comfort zones during the interview.

The experiences of confronting the end of one’s own life and the effects of the confrontation may provide insight into the determinants of general well-being for those subjectively nearing death. According to previous research, spiritual care may be a key aid in meeting existential needs; this includes being respectful, honest conversation, attention to overall well-being, and generally feeling affirmed and valued [18, 56], and can be provided by anyone [56]. The present interview study confirms that talking to one’s next of kin or familiar persons may also ease worries regarding this situation. The participants’ statements of gaining new insights as a perceived benefit of the research participation additionally highlight the positive effects of confronting this sensitive topic, even if only addressed within the research interview. Providing support may enable people who are distressed without realizing it to become aware of the roots and extent of their issues [18]. Even in healthy adults, spiritual interventions that focus on developing one’s own perspective on death increase happiness and quality of life [57]. However, our participants stated that they hardly used any professional services; in fact, such services were not even known about, and were therefore not accessible.

Since needs and preferences at the end of life are highly individual and malleable, support and care systems need to be accessible and flexible and offered as an invitation [17, 58, 59]. Patients need to be aware where they can seek assistance supported by health care providers or non-professionals who feel comfortable to engage in conversations about end-of-life issues such as dignity, meaning, autonomy and relationships [10, 18]. As such, awareness of spiritual and psychosocial needs among this population requires to be raised in order to provide customized support. The public support system also needs to be expanded, particularly with regard to the subjective perception among members of this group that they are a burden to the family members that assist them, which was also reported to be a major concern [60, 61]. An equivalent focus must also be applied to general social sensitization for public discourse that seeks to foster a strong culture of caring with respect to existential questions. Firstly, concepts relating to death and dying need to be included in the educational curriculum [62] within the field of healthcare. Therefore, the needed skill sets should be explored to inform training development. International guidelines to promote the field of handling existential issues within spiritual care inspired by existing guidelines like the National Consensus Project Guidelines [38] could expedite the introduction of existential topics within the care system and give caregivers security in practical application [31]. Secondly, more public relations work and low-threshold opportunities for confronting topics relating to death and dying need to be launched in order to draw public attention. To develop a profound knowledge base on the foundation of existing evidence community workshop and training programs should be expanded to develop competencies in spiritual and psychosocial care [63].

### Strengths and limitations

The participants were recruited from a variety of settings and different backgrounds, thus providing diverse experiences and views. With these interviews, we managed to open up a space for intimate and sensitive topics, and the respondents felt comfortable in spite of the emotional strain. Due to the first author and interviewer’s proximity to the data, it was particularly important to ensure that said data was analyzed independently with the second author in order to ensure that none of the potential insights were overlooked.

However, the selection process did impose limitations, as potential participants were fully informed regarding the nature of the study and the topics they would be asked to elaborate on. As such, it can be assumed that the study sample consisted of people who would feel comfortable sharing their views and attitudes toward death, and were unlikely to avoid death-related topics. The life-limiting circumstances of those in the sample group were related exclusively to cancer diseases, and participants in this group had an average age of 75 years. As a result, the possibility of an age and generation effect cannot be ruled out.

Since this qualitative type of study is based on a limited sample size, it does not seek to generalize results, but instead strives for an understanding of the particular context and to generate explanatory hypotheses. The transferability to similar contexts of existential discomfort is conceivable.

Furthermore, specific characteristics regarding economic status, educational background or religious beliefs were not pursued among the participants, as they were assumed to be of no significance. The sample represented the individuals to whom the results apply and matter, but it remains unclear whether the sample is representative of a larger population.

The Sars-CoV-2 pandemic impeded both the recruitment process and the conducting of the interviews, mainly due to restrictions on visitors. Nevertheless, it was possible to continue the project with the help of partners in practices and additional precautions and the finding of this study were not diminished or altered by the pandemic circumstances.

## Conclusion

The present study suggests that self-determination, a sense of dignity, and connection are all strengthened by confronting the finitude of one’s own life, and may work as mechanisms of action for greater well-being. Pro-active coping processes tend to move a concern, distress or problem toward alleviation, or even resolution. As such, the act of confronting existential questions regarding life and death and the positive effects of this act should be incorporated into professional and informal support frameworks, and access to social and healthcare services, which is currently fragmented needs to be improved upon. This request applies not only to people with severe diseases, but also to those who feel an urge to talk about existential life issues, such as people aged 80 and over, or perhaps even younger.

Future research should focus on developing, implementing and evaluating supportive interventions that aim to increase psychosocial and spiritual well-being for people who are subjectively nearing death. A differentiation between life-limiting diseases other than cancer and changes in attitudes towards confronting the end of one’s life across one’s entire lifespan should be considered.

## Supplementary Information

**Additional file 1.** Themes and codes derived from the data.

## Data Availability

The datasets generated and/or analyzed during the current study are not publicly available due to limitations of ethical approval involving the patient data and anonymity but are available from the corresponding author on reasonable request.
