# Supplementary material for: “My life became more meaningful”: confronting one’s own end of life and its effects on well-being—a qualitative study
Source: BMC Palliat Care. 2022 Apr 29;21:58. doi: 10.1186/s12904-022-00950-3 (PMC9050349; doi:10.1186/s12904-022-00950-3)
Supplement: Supplementary file 1 — Additional file 1. Themes and codes derived from the data. [file 12904_2022_950_MOESM1_ESM.pdf]

Additional file 1: Themes and codes derived from the data

Perspectives on the course of one's life	Effects of confronting finitude
Negative emotions	Process of normalization
Taking stock of one's life	Relief
Uncertainty	Autonomy and self-determination
Appropriateness/other orientation	Positive feelings
Satisfaction	Threatening ideas/negative feelings
Self-efficacy and sense of self-worth	Neutral/indifferent
Feeling of injustice	Assurance and orientation
Experiences with the concept of the end of life	Wishes toward approaches for confronting
Learned taboo on finitude	Therapeutic setting
Wartime experiences	Personalized services
Conversations and descriptions	Opportunities to talk
Repression	Sars-CoV-2
Grief and painful memories	Isolation
Feelings/attitude toward death and dying	No/hardly any confrontation
One's own death	Effects on attitude toward death
Thoughts of ending one's life/desire to do so	Positive effects
Insecurity	Social importance
Presence and awareness	Fear of infection or death
Oppressive	Limitations on everyday life
Rejection and distraction	Close relatives
Acceptance	Requesting support for people close to oneself
Fearful and unfamiliar	Concerns about people close to oneself
Relief/redemption	Peaceful environment
Being on one's own	Relationships and trustworthiness
Death of others	Wishes for the end of one's life
Mourning and painful	Physical and cognitive functions
Relief/redemption	Funeral
Death in general	Meaning in life
No confrontation	Peaceful and sudden death
Natural process/pragmatic	Independence and autonomy
Confronting end of life is necessary	Generativity
Hereafter	Dignity
Accompanying dying	Social connectedness
Deep interpersonal connection	Feeling like a burden
Profound and existential experiences	Healthcare
Focus on essential things in life/changing priorities	Arranging one's estate
Before dying	Everyday life activities
Planning for deterioration	Psychosocial comfort
Acute situation	Close relatives
Charitable actions	Requesting support for people close to oneself
Experiencing positivity	Concerns about people close to oneself
Getting things done	Peaceful environment
Social events and contact	Relationships and trustworthiness
Active confrontation with finitude	
Theoretical education	
Talking about death-related topics	
Preparing for death	
Personal reflection and thoughts	
Spirituality and faith	
Initial thoughts	
